# Proteasome inhibition in cancer is associated with enhanced tumor targeting by the adeno‐associated virus/phage

**DOI:** 10.1016/j.molonc.2012.08.001

**Published:** 2012-08-21

**Authors:** Justyna M. Przystal, Eloho Umukoro, Charlotte A. Stoneham, Teerapong Yata, Kevin O'Neill, Nelofer Syed, Amin Hajitou

**Affiliations:** ^1^ Division of Brain Sciences, Hammersmith Hospital Campus, Department of Medicine, Imperial College London, Burlington Danes Building, 160 Du Cane Road, London W12 0NN, United Kingdom; ^2^ Division of Brain Sciences, Charing Cross Campus, Department of Medicine, Imperial College London, London, United Kingdom

**Keywords:** Proteasome, Tumor targeting, Combination therapy, RGD, Bacteriophage

## Abstract

Bacteriophage (phage), which are viruses that infect bacteria only, have shown promise as vehicles for targeted cancer gene therapy, albeit with poor efficiency. Recently, we generated an improved version of phage vectors by incorporating cis genetic elements of adeno‐associated virus (AAV). This novel AAV/phage hybrid (AAVP) efficiently delivered systemically administered therapeutic genes to various tumor targets by displaying an integrin tumor‐targeting ligand on the phage capsid. However, inherent limitations in bacteriophage mean that these AAVP vectors still need to be improved. One of the limitations of AAVP in mammalian cells may be its susceptibility to proteasomal degradation. The proteasome is upregulated in cancer and it is known that it constitutes a barrier to gene delivery by certain eukaryotic viruses. We report here that inhibition of proteasome improved targeted reporter gene delivery by AAVP in cancer cells in vitro and in tumors in vivo after intravenous vector administration to tumor‐bearing mice. We also show enhanced targeted tumor cell killing by AAVP upon proteasome inhibition. The AAVP particles persisted significantly in cancer cells in vitro and in tumors in vivo after systemic administration, and accumulated polyubiquitinated coat proteins. Our results suggest that the proteasome is indeed a barrier to tumor targeting by AAVP and indicate that a combination of proteasome‐inhibiting drugs and AAVP should be considered for clinical anticancer therapy.

## Highlights

► Proteasome in cancer is a barrier to tumor targeting by RGD4C/AAVP.
► Proteasome inhibiting drugs enhance targeted gene transfer by RGD4C/AAVP.
► Proteasome inhibition increases targeted cancer gene therapy by RGD4C/AAVP.
► RGD4C/AAVP persistence in cancer is improved by proteasome inhibition

## 1 Introduction

Cancer gene therapy has faced a problem common to all forms of gene therapy, namely the lack of a systemically‐administered, safe and efficient vector to deliver the gene of interest at the diseased site. Animal viruses have been shown to be capable of ligand‐targeted gene delivery, but they require the elimination of their native tropism for mammalian cells so that they can be re‐targeted at alternative receptors (Hajitou et al., 2006a; Hajitou, 2010), which results in reduced efficacy (Allen et al., 2006; Ghosh and Barry, 2005; Hajitou et al., 2006a; Hajitou, 2010). Incorporation of targeting peptides derived from *in vivo* phage display screenings into viral vectors has been attempted, but with little success because either the ligand destroys the vector or the vector destroys the ligand (Ghosh and Barry, 2005; Hajitou, 2010). A solution to this limitation may be to use bacteriophage as the gene delivery vehicle (Hajitou et al., 2006a; Hajitou, 2010; Larocca et al., 1998; Poul and Marks, 1999). This eliminates the need to transfer peptides from phage display to a eukaryotic virus, and no native tropism for mammalian cells needs to be circumvented. Bacteriophage are safe and can be targeted by a ligand displayed on their capsid to a specific mammalian receptor after systemic administration (Hajitou, 2010). Unfortunately, phage particles are considered to be poor vectors, nevertheless, as they have evolved to infect bacteria only and, therefore, have no intrinsic strategies for delivering genes to mammalian cells.

To overcome this limitation, we have recently generated an improved version of such phage‐based vectors as hybrids between two single stranded DNA viruses; adeno‐associated virus (AAV) and M13 phage (termed AAV/Phage; AAVP). Within this novel vector, a targeted phage capsid serves as a vehicle to deliver a recombinant rAAV mammalian DNA cassette incorporated into an intergenomic region of the bacteriophage genome (Hajitou et al., 2006b). This vector showed superior gene delivery compared to a regular phage vector with long‐term gene expression *in vivo* after systemic delivery (Hajitou et al., 2006b). We found that this improved mammalian transduction efficiency is associated with several factors: the improved fate of the delivered gene through maintenance of the entire mammalian transgene cassette, better persistence of episomal DNA, and formation of concatamers of the AAV transgene cassette (Hajitou et al., 2006b, 2007). In these previous studies, we used AAVP displaying the cyclic RGD4C (CDCRGDCFC) peptide ligand to target overexpressed α_v_ integrins in tumors. Therapeutic genes were successfully delivered to the tumor site in mice and rats while sparing the normal organs after intravenous administration (Hajitou et al., 2006b, 2007, 2008; Tandle et al., 2009; Trepel et al., 2009). A recent study carried out under the direction of the National Cancer Institute of the USA has elegantly confirmed the potential of this technology (Paoloni et al., 2009). Targeted AAVP was used to deliver a cytokine, tumor necrosis factor‐α (TNFα), to cancers diagnosed in pet dogs. Repeated doses proved safe and resulted in complete eradication of aggressive tumors in some of these dogs (Paoloni et al., 2009).

It is clear that AAVP represent a new generation of phage‐based vectors that have promise. However, due to inherent limitations of bacteriophage, they need to be improved to enable them to overcome intracellular barriers in mammalian cells. Phage internalization assays have shown that 100% of cells *in vitro* internalize the targeted phage via a receptor‐mediated endocytosis, only as few as 10% of cells actually express the transgene (Hajitou et al., 2007). This is probably due to the fact that, unlike eukaryotic viruses, bacteriophage have no strategies to evade the barriers to infective agents that mammalian cells present. Proteasomes are one of these barriers. They are multi‐subunit enzymes responsible for the degradation of many cytosolic proteins (e.g. misfolded proteins, cyclins, and transcription factors) and for processing foreign proteins prior to the deployment of cellular immune responses (Groll et al., 1997; Kisselev, 2008; Tanaka et al., 2012). Numerous previous studies have reported the proteasome as an obstacle to some eukaryotic viral vectors (Monahan et al., 2010). Furthermore, cancers possess an elevated level of proteasome activity (Chen and Dou, 2010; Kisselev, 2008; Wu et al., 2010). It would therefore seem likely that the activity of proteasomes represents one possible barrier to the efficient delivery of AAVP vectors to cancer cells.

We report here an investigation of the efficacy of targeted gene delivery by RGD4C/AAVP to cancer in the presence of the proteasome inhibitors, MG132 and the Calpain 1 inhibitor LLnL. The MG132 and LLnL are peptide aldehyde inhibitors that reversibly inhibit the 26S proteasome activity (Kisselev and Goldberg, 2001; Lu et al., 2006; Masdehors et al., 2000; Vinitsky et al., 1992), and most widely used in proteasome inhibition studies (Gartel, 2010; Granot et al., 2007; Lu et al., 2006). We found that combination of proteasome inhibitors with RGD4C/AAVP resulted in significantly improved reporter gene expression *in vitro* and *in vivo*, and better tumor cell killing than the vector alone. Next, we established that this improved efficacy is associated with better persistence of the AAVP particles both *in vitro* and *in vivo* and with increased polyubiquitination of the AAVP coat proteins, when used in combination with a proteasome inhibitor. Our results strongly suggest that supplementary proteasome inhibition should be considered as the potential of AAVP vectors is further explored.

## 2 Material and methods

### 2.1 Reagents and cells

The Human Embryonic Kidney (HEK293) cell line was purchased from American Type Culture Collection (ATCC). Human M21 Melanoma cells were a gift from Dr David Cheresh (University of California, La Jolla), the human U87 glioblastoma cells were from Cancer Research UK and rat 9L glioblastoma cells were provided by Dr Hrvoje Miletic (University of Bergen, Norway). All these cell lines were maintained in Dulbecco's Modified Eagle's Medium (DMEM, Sigma) supplemented with 10% Fetal Bovine Serum (FBS, Sigma), Penicillin (100 units/ml, Sigma), Streptomycin (100 μg/ml, Sigma) and l‐Glutamine (2 mM, Sigma). Cells were cultured in a humidified atmosphere of 37 °C in a 5% CO_2_ and passaged every 3–4 days when they reached 80–90% confluence. To assess tumor cell killing *in vitro*, cells were incubated with medium containing ganciclovir (GCV) at 20 μM. GCV was renewed daily and cells were counted using the trypan blue‐exclusion methodology.

### 2.2 MTT assay

Mitochondrial activity (a measure of cellular viability) was measured with the MTT (3,4,5‐dimethylthiazol 2,5 diphenyltetrazolium bromide) assay and was used to determine the viability of cells following treatment with Z‐Leu‐Leu‐Leu‐al (MG132, Sigma) and the Calpain 1 inhibitor N‐Acetyl‐Leu‐Leu‐Norleu‐al (LLnL, Sigma). Cells were plated at a density of 4 × 10^3^ cells/well in a 96 well plate (Nun C). Stock solutions of 4 mM for MG132 and 40 mM for LLnL were prepared by using dimethyl sulfoxide (DMSO, Sigma). Cells were treated with varying concentrations of MG132 or LLnL. Complete medium (100 μl) containing the MG132 or LLnL drug was added to cells in triplicates. After 24 h, the proteasome inhibiting drug‐containing medium was removed and replaced with 100 μl of fresh medium. MTT assay was carried out 48 h later following manufacturer's instructions.

### 2.3 Vector production, purification and titration

We used the previously reported AAVP vector in which a cytomegalovirus (*CMV*) promoter drives a mammalian expression cassette containing reporter or therapeutic transgenes (Hajitou et al., 2006b). Targeted and non‐targeted phage viral particles were amplified, isolated and purified from the culture supernatant of host bacteria (*Esherichia coli* K91), as we described (Hajitou et al., 2007). Phage particles were sterile‐filtered through 0.45 μm filters, then titrated by infection of K91 host bacteria followed by colony counting on Luria–Bertani agar plates and expressed as transducing units (TU/μl) as described (Hajitou et al., 2007).

### 2.4 Cell transduction by vectors

Cell transduction by AAVP vectors was performed as we recently described in our detailed protocol (Hajitou et al., 2007). Briefly, cells were counted, plated in 48‐well plates and grown for 48 h to reach 60–80% confluence. Next, vectors were incubated with cells for 4 h in a 0.1 ml total volume of serum‐free medium with a ratio of 10^6^ TU vector per cell at 37 °C, followed by a medium change to medium plus 10% serum. Typically, non‐targeted vectors (without ligand) served as negative controls for the ligand‐targeting experiments. When indicated, treatment with the proteasome inhibitors MG132 or LLnL initiated during vector transduction and continued for 24 h. Analysis of cell transduction efficacy *in vitro* by the targeted vectors was carried out by using the green fluorescent protein (*GFP*) as well as the firefly luciferase (*Luc*) reporter transgenes. GFP expression by cells was monitored daily by using a Nikon Eclipse TE2000‐S fluorescence microscope that has a fitted Nikon digital camera (DXM1200F). GFP positive cells were counted and expressed as average of five fields of view. Luciferase reporter transgene expression in AAVP‐transduced cells was determined by using The Promega Steady‐glo^®^ luciferase assay kit following the manufacturer's protocol and quantified using a Promega plate reader, then normalized to 1 μg protein levels, determined by the Bradford assay. In addition, all the data were normalized to non‐targeted vectors.

### 2.5 Determination of tumor cell killing *in vitro*

U87 cells were seeded in a 48 Well‐plate and incubated for 48 h, to reach 60–80% confluence. Next, cells were transduced with targeted or non‐targeted AAVP‐*HSVtk*, carrying the *Herpes simplex virus thymidine kinase (HSVtk)* gene, in the presence or absence of the proteasome inhibitor MG132 as described above. GCV was added to cells (20 μM) at day 3 post vector transduction and renewed daily. Viable cells were counted at 48 h and 72 h post GCV treatment by using the trypan blue‐exclusion methodology.

### 2.6 Immunofluorescence

M21 melanoma cells were seeded on 18 mm^2^ coverslips in 12‐well plates. The next day, cells at approximately 50–60% confluency were incubated with phage in the presence or absence of MG132 for 4 h in serum free medium, then overnight in complete medium as described above. Next, cells were washed with phosphate buffered saline (PBS) and fixed in PBS containing 4% paraformaldehyde. Cells were then incubated for 5 min in 50 mM Ammonium Chloride to quench free aldehyde groups from fixation, permeabilized with 0.2% Triton X‐100, washed, and blocked with PBS containing 2% BSA. Subsequently, cells were incubated with rabbit anti‐M13 bacteriophage, that binds specifically to phage coat proteins of M13 phage, (diluted 1:1000, Sigma) and mouse anti‐ubiquitin (1:200, Invitrogen) antibodies for 1 h at room temperature followed by 1 h incubation with anti‐rabbit and anti‐mouse AlexaFluor‐conjugated secondary antibodies (diluted 1:750, Invitrogen). Cells were washed three times in PBS and twice in distilled water, allowed to air‐dry and mounted in the presence of DAPI (1:2000, Sigma) in Mowiol mounting medium (prepared in‐house). Images were acquired with a Leica laser scanning confocal microscope.

### 2.7 Recovery of internalized phage particles from cancer cells *in vitro*

*In vitro*, U87 cells were seeded in 48‐well plates and grown until 60–80% confluent. The cells were then incubated with targeted or control non‐targeted vectors with or without MG132 for 4 h. In MG132‐treated wells, the medium was replaced with complete medium containing the MG132 drug. After 24 h, the plates were placed on ice for 5 min to stop phage internalization, then cells were washed 3 times with 1X PBS. Cell surface AAVP phage particles were inactivated by subtilisin treatment (4.5 mg/ml) for 15 min at room temperature, then subtilisin was inactivated with EDTA (2 mM). Internalized AAVP particles were obtained by treating cells with the lysis buffer (2% deoxycholic acid, 2 mM ethylene diamine tetraacetic acid, and 10 mM Tris [pH 8.0]) for 1 h at room temperature. The number of AAVP particles were counted by using k91Kan bacterial infection and counting transducing units (Tandle et al., 2009).

### 2.8 Animal models

The *in vivo* experiments were carried out in adherence to the UK Coordinating Committee on Cancer Research (UKCCCR) guidelines for the Welfare of Animals in Experimental Neoplasia and according to the institutional and Home Office guidelines. Mice were anesthetized by gas (2% isoflurane and 98% O_2_) inhalation. To establish subcutaneous tumors in mice, a total of 1 × 10^7^ U87 or 4 × 10^6^ M21 cells were subcutaneously implanted into immunodeficient nude mice. When tumors reached the required volumes, tumor‐bearing mice received a single intravenous dose, (5 × 10^10^ TU/mouse), through the tail vein of targeted RGD4C/AAVP or control non‐targeted AAVP. MG132 (2.5 μg/g body weight) was administered to mice by intraperitoneal injection. In phage recovery experiments, xenografts were removed after ∼18 h, weighed, followed by tumor grinding and incubation of the tumor tissue homogenates with K91 host bacteria for recovery of integral and infectious phage particles that were quantified as TU normalized to the tumor weight. We have repeated the experiments twice and used 4 to 5 mice per group in order to reduce animal suffering and apply the 3Rs (“Reduce, Refine and Replace”) in accordance with the institutional and Home Office guidelines.

### 2.9 *In* vivo bioluminescence imaging

To monitor *Luc* transgene expression in the whole living animals and measure *Luc* expression in tumors after intravenous administration of AAVP‐*Luc* carrying the *Luc* reporter gene, we used subcutaneous M21‐derived tumors in immunodeficient mice. Tumor‐bearing mice were anesthetized and administered with 100 mg/kg of d‐luciferin (Gold Biotechnology) by subcutaneous injection into the loose skin over the neck. Photonic emission was imaged over a time course by using the *In Vivo* Imaging System (IVIS 100) (Caliper Life Sciences). Regions of interest (ROI) were defined manually over the tumors for measuring signal intensities recorded as total photon counts per second per cm^2^ (p/sec/cm^2^/sr) within ROI. Similar color scale bar was applied for all representative images.

### 2.10 Statistics

Statistical analyses were performed by using GraphPad Prism software (version 5.0). Error bars represent standard error of the mean (s.e.m). *P* values were generated by ANOVA and denoted as follows: *p < 0.05, **p < 0.01 and ***p < 0.001.

## 3 Results

### 3.1 The proteasome is a barrier to gene delivery by the α_v_ integrin‐targeted RGD4C/AAVP

As proof of concept, we first wanted to determine whether the proteasome represents a barrier to targeted gene delivery by RGD4C/AAVP. Thus, we transduced the α_v_ integrin‐expressing HEK293 cells with RGD4C/AAVP vector and assessed the effect of MG132. We chose the HEK293 cell line for initial experiments because these cells have extensively been used as a standard *in vitro* model to characterize cell transduction by RGD4C/AAVP since they express high levels of α_v_β3 and α_v_β5 integrins (Hajitou et al., 2006b; Hajitou et al., 2007). Also, despite the presence of these integrin subunits in the HEK293 cell line, RGD4C/AAVP mediates only very modest transgene expression in these cells. Therefore, it is possible that the vector is able to access the cell cytoplasm and efficacy may be reduced by proteasomal degradation. In order to assess whether gene transfer efficiency by AAVP is reduced by proteasome activity, the efficacy of transduction was evaluated in the presence of increasing concentrations of MG132. HEK293 cells were incubated with RGD4C/AAVP or control non‐targeted vector bearing the *GFP* or *Luc* reporter genes. The results showed that efficiency of gene‐transfer by RGD4C/AAVP was strongly enhanced overtime by the addition of the proteasome inhibitor. For instance, at day 7 post‐transduction, when a maximum of transgene expression is achieved in HEK293 cells by RGD4C/AAVP, treatment with 5 μM MG132 resulted in increased number of GFP‐positive cells as shown by fluorescence images of RGD4C/AAVP‐mediated GFP expression in HEK293 cells (Supplementary Figure 1A). This data was confirmed with the quantitative analysis of *Luc* activity that showed a ∼2.1‐fold increase of RGD4C/AAVP‐mediated *Luc* expression in MG132‐treated cells over non‐treated cells (Supplementary Figure 1B). Importantly, no *Luc* activity was detected in cells that received non‐targeted vector either alone or in combination with MG132 (Supplementary Fig. 1B).

### 3.2 Sensitivity of cancer cells to MG132 and LLnL

**Figure 1:**
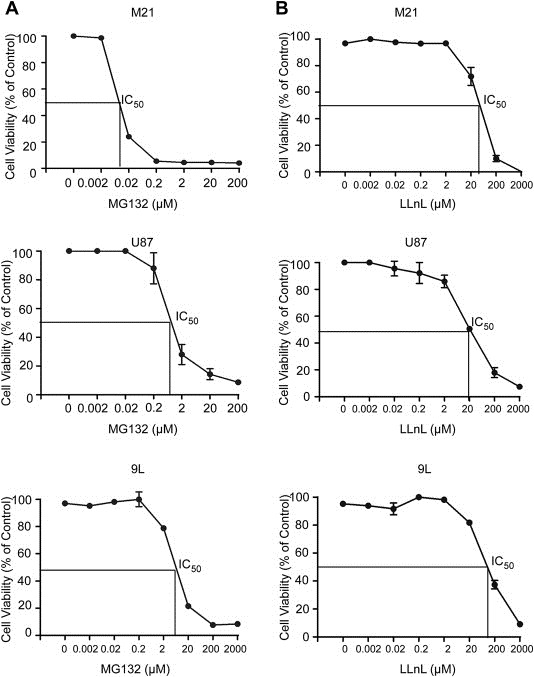
Cytotoxicity of the proteasome inhibitors MG132 and LLnL on cancer cell lines. The human M21 melanoma cells as well as the human U87 and rat 9L glioblastoma cells were cultured in 96‐well plates, then treated with increasing concentrations of MG132 (A) or LLnL (B) ranging from 0.002 μM to 2000 μM for 24 h. Subsequently, cells were grown for further 48 h without the drug. Cell survival was determined by using the MTT assay and expressed as percentage of cells counted in parallel cultures without the drug. The continuous lines indicate the estimated IC50 values. These assays were repeated twice in triplicate and the results shown are representative of one experiment.

After demonstrating that transgene expression by RGD4C/AAVP is clearly improved by inhibition of proteasome, we next sought to assess the effect of proteasome inhibitors on the efficacy of gene delivery by AAVP vectors in cancer cells. We conducted efficacy studies of proteasome inhibition on RGD4C/AAVP efficiency in the Human M21 melanoma cells known for their expression of the α_v_ integrin receptors for RGD4C ligand (Hood et al., 2002; Tandle et al., 2009). Additionally, to rule out the possibility that the observed effects of MG132 and LLnL are tumor specific, we analyzed efficacy of these drugs on the human U87 and rat 9L glioblastoma cells. First, we set to determine the sensitivity of cancer cells to increasing concentrations of MG132 and LLnL. Thus, the cytotoxicity of the drugs was investigated *in vitro* on M21, U87 and 9L cancer cell lines. Tumor cells were treated with various concentrations of MG132 or LLnL ranging from 0.002 μM to 2000 μM and compared to non‐treated cells. In all cell lines, cell survival in the presence of MG132 or LLnL decreased as the concentration of the drug increased (Figure 1). Unlike LLnL, cell viability assays showed that MG132 was more toxic on the M21 cell line than U87 and 9L cells as a rapid death of M21 cells (75%) already started at MG132 concentrations as low as 0.02 μM, while this dose induced a slight toxicity only on U87 and 9L cells (Figure 1A). Cytotoxic doses expressed as IC_50_ values, corresponding to inhibitory concentration required to reduce the cell survival by 50%, are shown by the lines on the graphs to approximate the IC_50_ value (Figure 1). We found that 50% of cell death in the presence of MG132 was induced by ∼0.01 μM in M21 cells, while in U87 cells and 9L, 50% of cell death was obtained with 1 μM and 5.733 μM of MG132, respectively (Figure 1A). The IC_50_ of LLnL were 48.65 μM for M21 cells, 18.58 μM for U87 and 112.2 μM for 9L cells (Figure 1B). MG132 concentrations used in subsequent experiments to assess effect on gene delivery by AAVP vector ranged below the IC_50_ and cause little to no toxicity. Accordingly, we selected MG132 concentrations of 0.0035 μM for M21 cells, 0.3 μM for U87 and 0.2 μM for 9L cells. In contrast, the concentrations of LLnL tested in following experiments were markedly higher than that of MG132 and reached 10 μM for M21, 20 μM for U87 and 2 μM for 9L cells.

### 3.3 Inhibition of proteasomes increases reporter gene delivery by RGD4C/AAVP in cancer cells *in vitro*

**Figure 2:**
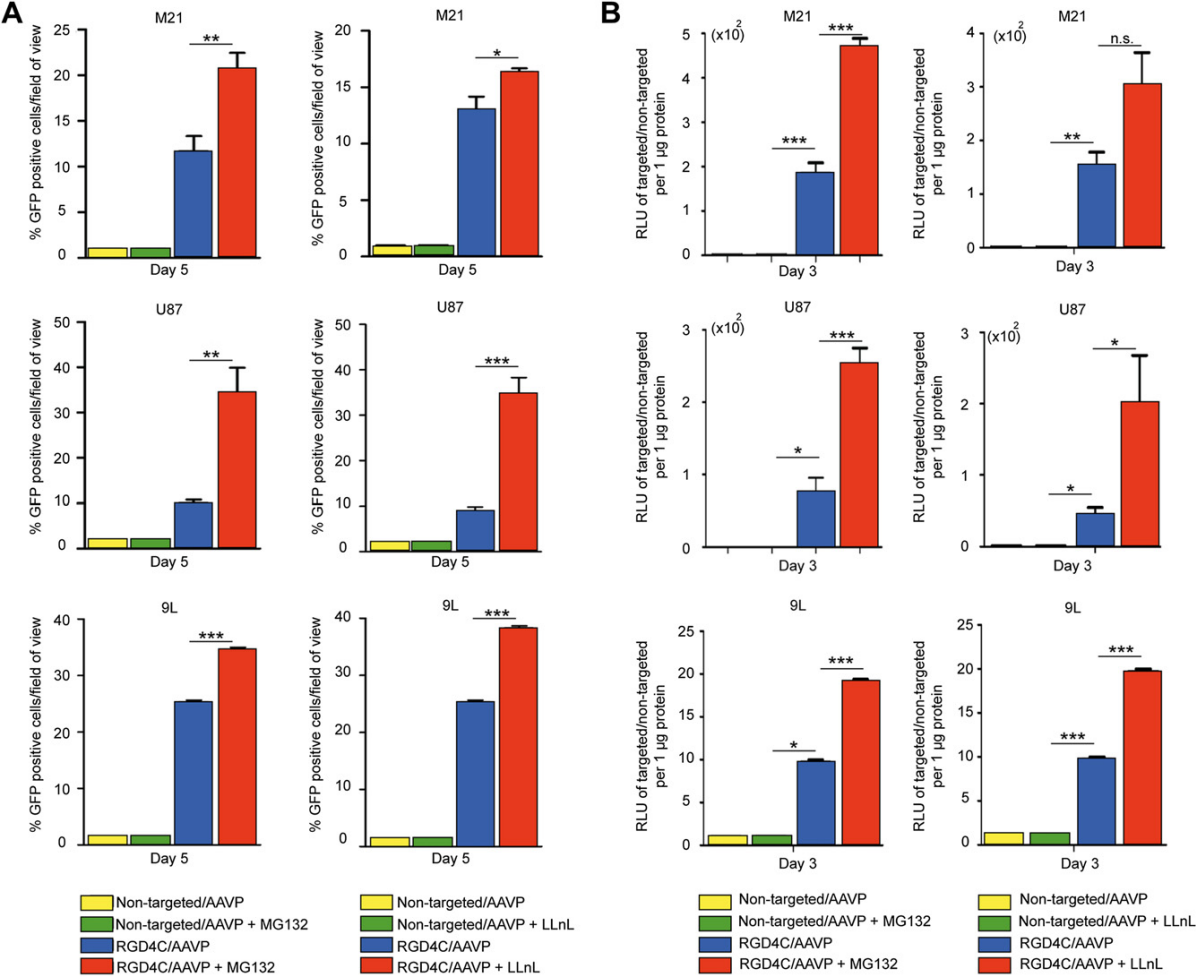
MG132 and LLnL increase targeted gene delivery by RGD4C/AAVP to cancer cells in vitro. The M21, U87 and 9L cells (60–80% confluent in 48‐well plates) were incubated with targeted RGD4C/AAVP‐GFP, RGD4C/AAVP‐Luc, or control non‐targeted vectors for 4 h in serum‐free medium in the presence of 0.0035 μM, 0.3 μM and 0.2 μM of MG132 or 10 μM, 20 μM and 2 μM of LLnL, respectively. Subsequently, MG132‐ and LLnL‐treated cells were grown in complete medium containing MG132 or LLnL for 24 h. A) Quantification of GFP positive cells in M21, U87 and 9L cell lines. GFP expression was monitored at day 5 post vector transduction and expressed as average of GFP positive cells in five fields of view. Cell transduction experiments in the absence of MG132 and LLnL were included as controls. All experiments were repeated twice in triplicate and results shown are representative of one experiment. B) Quantitative analyses of Luc‐mediated reporter transgene expression by AAVP vector in M21, U87 and 9L cells. Cell transduction experiments in the absence of MG132 or LLnL were included as controls. Luc measurement assay was performed at day 3 post vector transduction and normalized to protein concentration determined by the Bradford assay. Results represent the average relative luminescence units (RLU)/μg of protein from triplicate wells. All data were normalized to non‐targeted vector.

As an initial analysis of MG132 effect on efficiency of transgene expression in cancer cells, we transduced tumor cells with RGD4C/AAVP‐*GFP* vector carrying the *GFP* reporter transgene in the presence or absence of MG132, then counted and monitored GFP positive cells. The data showed that GFP expression was generally stronger over time in cells that received a combination of RGD4C/AAVP‐*GFP* and MG132 compared with cells treated with vector alone (Figure 2A left panel). For instance, at 5 days post‐vector transduction, we observed GFP expression in ∼21% of the M21 cells transduced by the RGD4C/AAVP‐*GFP* construct in the presence of MG132 compared to ∼11% of cells transduced by RGD4C/AAVP‐*GFP* vector alone (Figure 2A left panel). We also observed that ∼34% of the U87 and ∼35% of the 9L cells transduced by RGD4C/AAVP‐*GFP* had GFP expression in the presence of MG132 compared to only ∼11% and 25% of GFP positive cells transduced with RGD4C/AAVP‐*GFP* vector alone, respectively (Figure 2A left panel). Next, to confirm that RGD4C/AAVP and MG132 combination improves gene delivery in tumor cells, we carried out a quantitative analysis of transgene expression by using AAVP‐*Luc* vectors expressing the firefly *Luc* reporter gene. Consistently with GFP reporter transgene expression experiments, we observed a significant increase in RGD4C/AAVP‐mediated *Luc* expression by MG132 treatment compared to untreated and transduced cells (Figure 2B left panel). For instance, at day 3 post‐vector transduction, MG132 boosted Luc activity by ∼2.6‐, ∼3.5‐ and ∼1.97‐fold in RGD4C/AAVP‐transduced M21, U87 and 9L cells, respectively (Figure 2B left panel).

To confirm that the enhanced cancer cell transduction by RGD4C/AAVP in combination with proteasome inhibition is not unique to MG132, we investigated the effect of LLnL, as an additional proteasome inhibitor, and found that LLnL improves gene delivery to cancer cells by RGD4C/AAVP carrying either *GFP* (Figure 2A right panel) or *Luc* reporter genes (Figure 2B right panel). For example, at day 3 post vector transduction, quantitative analysis of *Luc* transgene expression showed that addition of LLnL resulted in ∼2.23 fold, ∼6.35 fold and ∼2.0 fold increase in Luc activity in M21, U87 and 9L cells, respectively (Figure 2B right panel). Finally, cells transduced with control non‐targeted AAVP vectors lacking the RGD4C ligand were also included in the experiments as negative controls. Importantly, no reporter gene expression was detected in cells that received non‐targeted AAVP alone or in combination with MG132 or LLnL (Figure 2A and B).

### 3.4 Proteasome inhibitor improves reporter gene delivery by RGD4C/AAVP in tumors *in vivo* upon systemic administration

**Figure 3:**
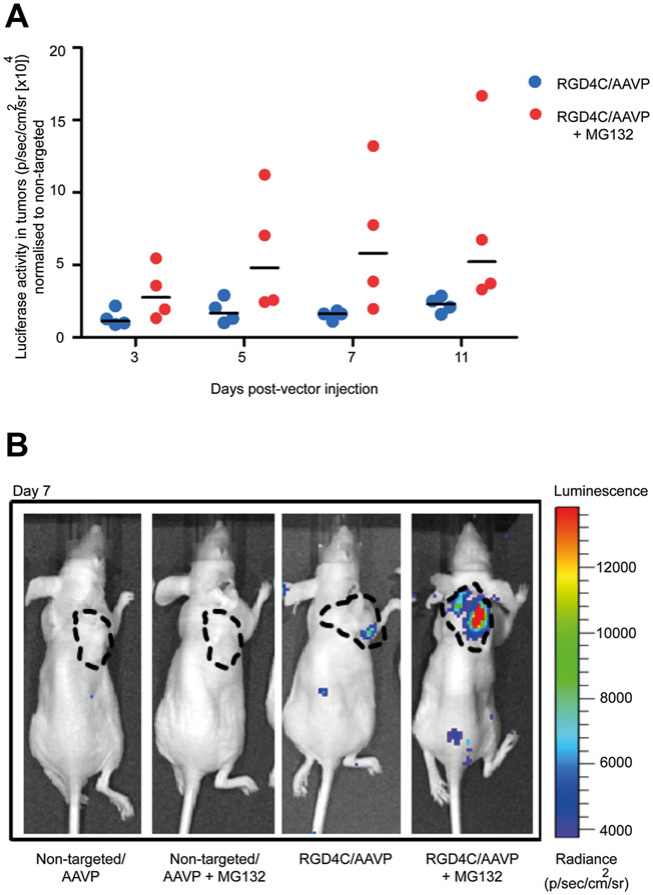
Efficacy of targeted systemic gene delivery in vivo by RGD4C/AAVP is increased in combination with MG132. In vivo bioluminescent imaging (BLI) of Luc expression. Immunodeficient nude mice bearing M21 subcutaneous xenografts (∼200 mm3) received a single intravenous dose (5 × 1010 TU/mouse) of the targeted RGD4C/AAVP‐Luc or control non‐targeted vector, alone or in combination with MG132 (2.5 μg/g body weight). Luc transgene expression was monitored at different days post vector administration. A) Serial real‐time quantification of Luc expression in individual tumors from tumor‐bearing mice treated with RGD4C/AAVP alone or in combination with MG132. Luc expression from targeted RGD4C/AAVP‐Luc was normalized to that of control non‐targeted vector. B) Representative tumor‐bearing mice from all the experimental groups at day 7 post vector administration. A standard calibration scale is provided.

Next, we evaluated the efficacy of targeted gene delivery to tumors after systemic administration of RGD4C/AAVP vector in combination with the proteasome inhibitor MG132, and compared this with vector alone. The MG132 drug has been commonly used for *in vivo* studies in mice (Grimes et al., 2005). We used a standard experimental setup for *in vivo* imaging of the *Luc* transgene reporter, and assessed the bioluminescence imaging (BLI) for noninvasive monitoring of temporal dynamics and spatial heterogeneity of *Luc* expression in living, tumor‐bearing mice. As an initial preclinical model, we used immunodeficient nude mice bearing subcutaneous tumors derived from M21 melanoma cells. Indeed, the M21‐derived tumor model in nude mice was previously used to assess efficacy of targeted tumor transduction *in vivo* by RGD4C/AAVP vector following intravenous administration of vector to tumor‐bearing mice (Tandle et al., 2009). Moreover, M21 tumor cells induce moderately growing tumors allowing repetitive imaging of mice over time without reaching large size tumors to avoid animal suffering. By using repetitive BLI, we visualized and quantitated *Luc* expression in M21‐derived tumors over a time course after a single systemic administration of RGD4C/AAVP‐*Luc*, or control non‐targeted vectors alone or in combination with MG132. Consistent with our previous reports (Hajitou et al., 2006b, 2007; 2008; Tandle et al., 2009), *Luc* expression within M21 tumors was detectable at day 3 after RGD4C/AAVP‐*Luc* administration (Figure 3A). Interestingly, enhanced tumor expression of *Luc* gene was achieved at all time‐points (days 3–11) by RGD4C/AAVP‐*Luc* when used in combination with MG132 as compared to RGD4C/AAVP‐*Luc* vector alone (Figure 3A). Finally, no tumor‐associated bioluminescent signals were observed in mice receiving control non‐targeted vectors alone or in combination with MG132 (Figure 3B).

### 3.5 Proteasome inhibition in tumor cells results in accumulation of polyubiquitination of AAVP coat proteins

**Figure 4:**
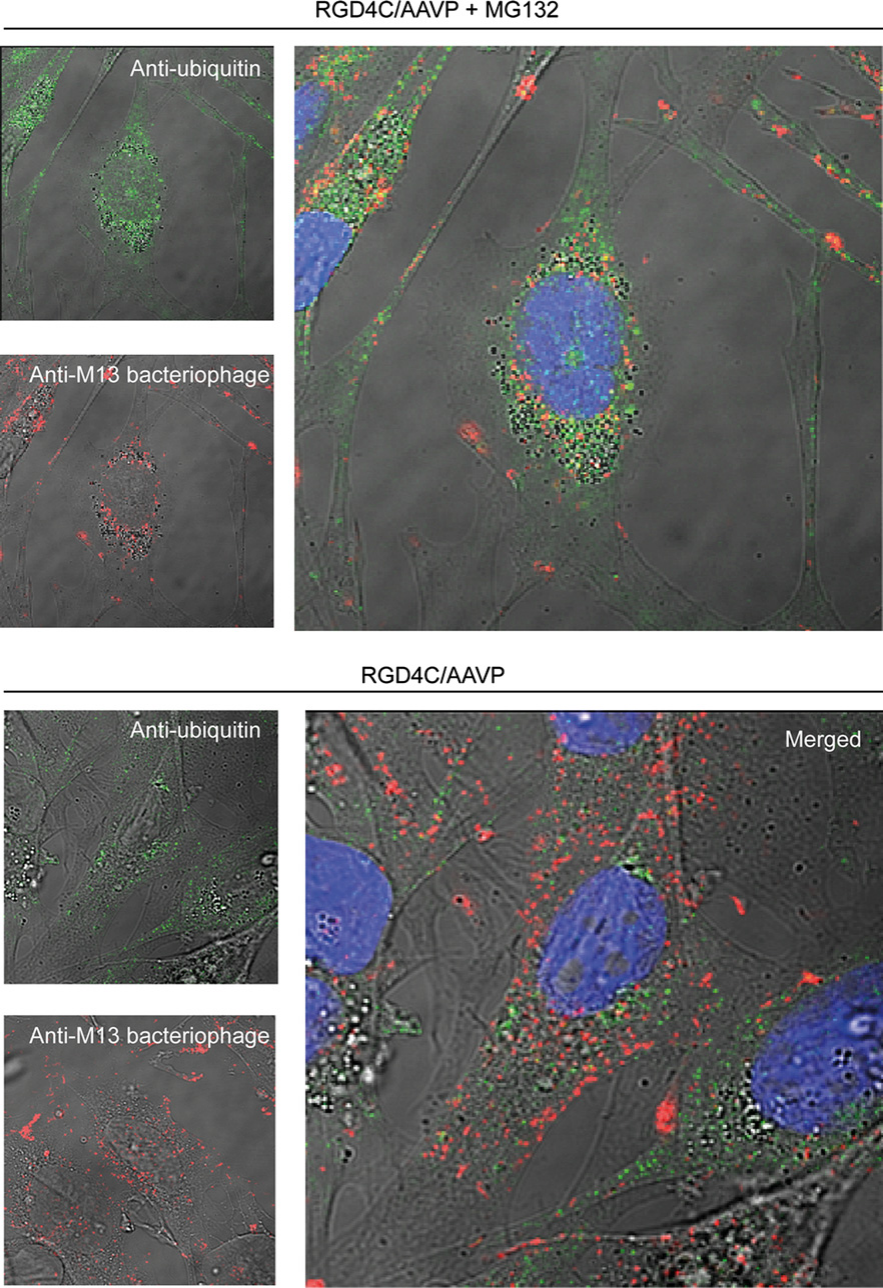
Polyubiquitination of RGD4C/AAVP particle is increased upon treatment with MG132. Colocalization of RGD4C/AAVP and ubiquitin was investigated by immunofluorescence double staining of RGD4C/AAVP and ubiquitin followed by confocal microscopic analysis. M21 melanoma cells were incubated in vitro with targeted RGD4C/AAVP or control non‐targeted vectors for 4 h in serum‐free medium in the presence or absence of MG132, followed by growth in complete medium. After 24 h, the RGD4C/AAVP was detected using rabbit anti‐M13‐phage primary and goat anti‐rabbit AlexaFluor‐594 secondary antibodies (shown in red) and ubiquitin was stained using mouse anti‐ubiquitin primary and AlexaFluor‐488 secondary antibodies (green). Representative single optical sections are shown.

The 26S proteasome mediates degradation of polyubiquitinated protein substrates labeled with polyubiquitin chains (Zhu et al., 2005). Thus, we sought to investigate whether inhibition of proteasome leads to increased polyubiquitination of AAVP phage coat proteins. We performed these experiments in the M21 cell line as these cells were previously used for microscopic analysis of intracellular RGD4C/AAVP (Tandle et al., 2009). M21 tumor cells were transduced with either targeted RGD/AAVP vector or non‐targeted control in the presence or absence of MG132. Subsequently, the cells were analyzed for co‐localization of AAVP particle proteins and ubiquitin by immunofluorescence as reported (Neumann et al., 2007), by using antibodies against ubiquitin and phage coat proteins (Figure 4). Confocal microscopic analyses showed strong co‐localization of ubiquitin and AAVP coat proteins in cells treated with MG132 (Figure 4). These data prove accumulation of polyubiquitination of the AAVP coat proteins in the presence of MG132.

### 3.6 MG132 increases tumor cell killing by RGD4C/AAVP*‐HSVtk* and GCV

**Figure 5:**
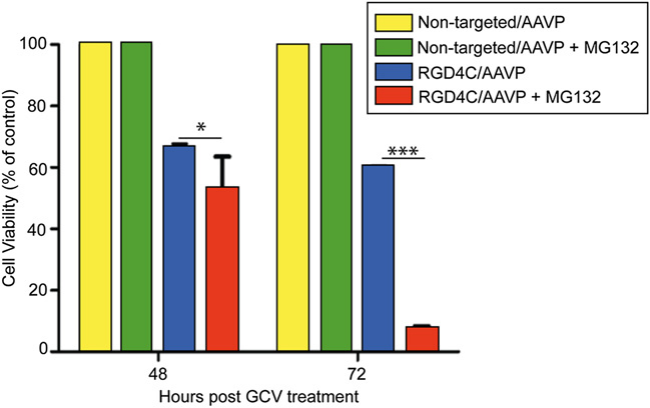
Effect of MG132 on cytotoxic gene therapy by RGD4C/AAVP in vitro. U87 glioblastoma cells grown in 48 well‐plates (60–80% confluent) were transduced with RGD4C/AAVP‐HSVtk or control non‐targeted vectors expressing the HSVtk gene in the presence or absence of MG132 as described above. Then cells were treated with GCV (20 μM) at day 3 post vector transduction, and renewed daily. Cancer cell killing was quantified at 48 h and 72 h post GCV treatment. Cells were counted by using the trypan blue‐exclusion methodology. Results were normalized to non‐targeted vector.

To test the tumor cell killing efficacy of the RGD4C/AAVP and MG132 combination *in vitro*, we constructed the RGD4C/AAVP‐*HSVtk* vector carrying the *HSVtk* gene. This gene can serve as a suicide gene when combined with GCV (Hajitou et al., 2006b; Trepel et al., 2009). We compared the RGD4C/AAVP‐*HSVtk* construct with the combination of RGD4C/AAVP‐*HSVtk* and MG132. We chose to conduct our experiments in the U87 human glioblastoma model since this tumor type is highly aggressive and remains a major challenge to treat in patients. The U87 tumor cells were transduced with targeted RGD4C/AAVP‐*HSVtk* or control non‐targeted vector in the presence or absence of MG132. *HSVtk* suicide gene therapy was induced at day 3 post‐vector transduction by treatment with GCV for 48 h or 72 h. As shown in Figure 5 and Supplementary Figure 2, addition of GCV resulted in significantly higher death of U87 cells treated with the combination of RGD4C/AAVP‐*HSVtk* and MG132 than that of cells transduced with RGD4C/AAVP‐*HSVtk* vector alone at both 48 h and 72 h post‐GCV treatment (Figure 5).

### 3.7 MG132 enhances persistence of the RGD4C/AAVP phage particles in cancer cells *in vitro* and in tumors *in vivo* after systemic administration

**Figure 6:**
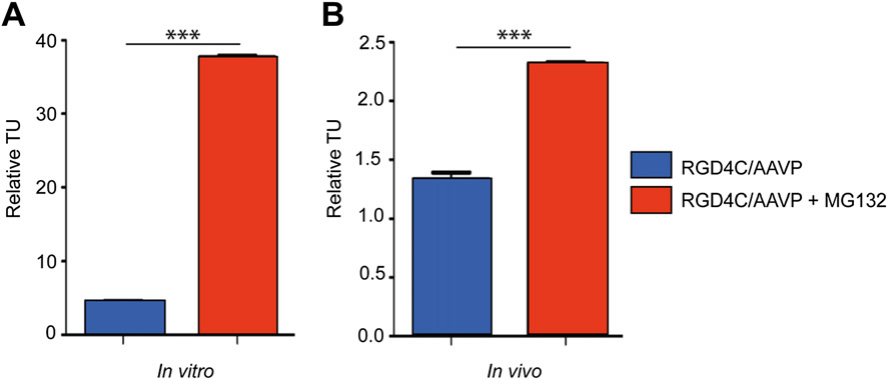
Persistence of RGD4C/AAVP phage particles upon treatment with MG132. A) Phage recovery in vitro. U87 gliobalstoma cells were incubated in vitro with targeted RGD4C/AAVP‐HSVtk or control non‐targeted vectors for 4 h in serum‐free medium in the presence or absence of MG132. Subsequently, cells were grown in complete medium containing MG132. After 24 h, cells were washed extensively with PBS and with subtilisin at 4.5 mg/ml. Phage viral particles were recovered by infection of K91 E.Coli. Results represent the mean phage TU/μl of triplicate wells ± s.e.m. B) Phage recovery in vivo. Immunodeficient nude mice with established U87 tumors (∼200 mm3) received a single intravenous dose of either targeted RGD4C/AAVP‐HSVtk (5 × 1010 TU) or control non targeted vector. Treatment with MG132 was simultaneously performed with vector and was administered intraperitoneal with (2.5 μg/g body weight). After 18 h, tumor‐bearing mice were killed, the tumors were harvested and targeted and control AAVP were recovered. Experiments were repeated twice. A representative experiment is shown. All data were normalized to non‐targeted vector.

To gain further insight into the improved efficacy of gene delivery and tumor cell killing by RGD4C/AAVP following proteasome inhibition, we next investigated the effect of MG132 on the persistence of the RGD4C/AAVP phage particles in U87 tumor cells *in vitro*. Thus, we carried out a recovery assay of internalized AAV/phage particles in the presence and absence of MG132. Intracellular and intact infectious phage was quantified by recovery from cell lysates followed by infection of host bacteria, and counting of transducing units. The data revealed that cells incubated with targeted RGD4C/AAVP showed significant phage internalization (Figure 6A), confirming that cell entry of vector is mediated through the RGD4C ligand. In sharp contrast, MG132 treatment of U87 cells transduced with the targeted RGD4C/AAVP resulted in a dramatic increase, ∼7 fold, of recovery of the integral and undamaged intracellular AAV/phage particles (Figure 6A).

Subsequently, we sought to investigate whether inhibition of proteasome *in vivo* can improve persistence of the phage particles in tumors after systemic administration of the RGD4C/AAVP vector to tumor‐bearing mice. Cohorts of mice bearing size‐matched subcutaneous tumors (∼200 mm^3^) received a single intravenous administration of RGD4C/AAVP or control non‐targeted vectors either alone or in combination with intraperitoneal administration of MG132. At ∼18 h after vector administration, tumor‐bearing nude mice were killed, and tumors were collected. Relative tumor targeted RGD4C/AAVP was quantified by recovery from tumor tissue homogenates, bacterial infection, and counting transducing units. Importantly, we observed a marked tumor homing of RGD4C/AAVP in U87 xenografts in combination with MG132 (Figure 6B). More specifically, we found ∼2 fold tumor enrichment of RGD4C/AAVP when administered simultaneously with MG132 compared with tumor homing of targeted RGD4C/AAVP alone (Figure 6B). These data further establish that proteasome inhibition improves the persistence of the RGD4C/AAVP *in vivo* in tumor‐bearing mice after systemic administration.

## 4 Discussion

Bacteriophage have been widely studied and have been safely administered both to adults and children over many years (Fortuna et al., 2008). Recently, the US‐FDA has even approved the use of some bacteriophage preparations as anti‐bacterial food additives, further supporting the safety of these agents (Lang, 2006). Our recent reports on the novel AAVP identify that these are promising vectors for targeted gene delivery for various diseases, including cancer. However, due to the low evolution profile of bacteriophage as bacteria viruses, the AAVP phage‐based vectors have to confront several barriers to accomplish effective gene delivery in mammalian cells. It is additionally widely accepted that human cancer cells possess elevated levels of proteasome activity, and are more sensitive to proteasome inhibitors than normal cells (Chen and Dou, 2010; Kisselev, 2008; Kisselev and Goldberg, 2001; Wu et al., 2010). Taking all of this evidence together, we hypothesized that our novel AAVP vector may have improved reporter gene delivery, and thus increased cancer cell killing, if it is used in conjunction with proteasome inhibitors.

Our initial studies in the HEK293 cells, an *in vitro* cellular model that has been used to characterize transduction by RGD4C/AAVP, indeed showed only modest gene expression even when using very high vector titers (Hajitou et al., 2007). This further supported the notion that our AAVP vector may be subjected to high levels of proteasomal degradation. We further investigated intracellular barriers to the RGD4C/AAVP vector in HEK293 cells and found that cell transduction in the presence of the proteasome inhibitor MG132 resulted in a strong increase in RGD4C/AAVP‐mediated gene transfer *in vitro*. We then confirmed that blocking the proteasome in various cancer cell lines results in increased reporter *GFP* and *Luc* transgene expression. These data are consistent with previous studies reporting an increase in GFP positive cells when transducing cells with lambda λ phage in the presence of MG132 (Volcy and Dewhurst, 2009). Importantly, BLI of Luc experiments showed that treatment of tumor‐bearing mice with MG132 boosts targeted gene delivery to tumors following intravenous administration of RGD4C/AAVP. This report is the first to show efficacy of proteasome inhibiting drugs on targeted systemic gene delivery to tumors *in vivo*. In our studies, the increased recovery of integral and undamaged intracellular and intratumoral AAVP particles after blocking the proteasome indicates the AAVP phage particles are in fact susceptible to proteasomal degradation. Moreover, the increased polyubiquitination of AAVP particle coat proteins upon treatment with MG132 further confirms proteasomal degradation of AAVP. Indeed, it well known that the 26S proteasome accomplishes degradation of proteins labeled with polyubiquitin chains (Glickman and Ciechanover, 2002). After recognition by the 19S proteasome regulatory complex, polyubiquitin chains are disassembled and substrates are processed in the 20S core of proteasome. Numerous studies have shown that MG132 increases protein polyubiquitination (Yan et al., 2011). It is important to note that other mechanisms of action of MG132 should also account for its enhancing effect on cell transduction efficiency by RGD4C/AAVP. For instance, MG132 was reported to alter intracellular trafficking of some viruses (Denby et al., 2005; Yu and Lai, 2005). However, while reports showed that MG132 enhances nuclear delivery of viruses such as AAV (Denby et al., 2005), other studies reported lysosomal accumulation of viruses in the presence of MG132 (Yu and Lai, 2005). In the present work, as well as in other studies (Souza et al., 2006), confocal microscopic imaging of intracellular bacteriophage shows that RGD4C/phage particles have discrete and major vesicular localization. Yet, we did not detect any visible alteration of the vesicular pattern of RGD4C/AAVP particles following MG132 treatment (Supplementary Figure 3). Recently, we found that the majority of RGD4C/AAVP is sequestered in lysosomal vesicles upon entry into cells and that the minor cytoplasmic RGD4C/AAVP is invisible with confocal or electronic microscopic imaging (unpublished data). The RGD4C/AAVP phage particles might also have transient cytoplasmic passage, or enter the cytoplasm in a broken or uncoated shape. It should also be noted, however, that MG132 has been reported to inhibit lysosomal cathepsins in addition to the proteasome (Tsubuki et al., 1996). Additionally, it was shown that MG132 increases coxsackie and adenovirus receptor expression in a colon cancer cell line resulting in enhanced adenovirus transfer, targeted gene expression and oncolysis (Zhang et al., 2008). To date, we constantly observed that association of improved AAVP transduction efficiency with any significant change of AAVP distribution or compartmentalization is unnoticeable by microscopic imaging. In particular, MG132 shows some toxicity resulting in decreased experimental efficiency (Caron et al., 2004).

Collectively, our studies confirm the proteasome appears to be a significant barrier to the efficacy of targeted gene delivery by RGD4C/AAVP. Although animal viruses have developed strategies to resist and protect their proteins against proteasomal degradation, the proteasome has been shown to be a barrier to gene delivery by a number of eukaryotic viruses such as lentiviral vectors (Santoni de Sio et al., 2008). Viruses such as adeno‐associated virus, also appear to undergo ubiquitination and degradation by the proteasome, and transgene expression by AAV vectors can be significantly enhanced in the presence of proteasome inhibitors (Jennings et al., 2005). It is understandable that phage, which has evolved to infect bacteria only, has no developed strategies to overcome the proteasome barrier in mammalian cells, which renders them susceptible to proteasomal degradation.

Our *in vitro* data with MG132 effect on efficacy of gene delivery by RGD4C/AAVP were also confirmed by using the LLnL as an additional proteasome inhibitor. However, the findings following analysis of the effect of LLnL on transgene expression by RGD4C/AAVP in M21 cells *in vitro* merit further discussion. Although, LLnL stimulated transgene expression by RGD4C/AAVP in the M21 cells, quantitative analysis of *Luc* expression revealed no significant difference between cells transduced with targeted RGD4C/AAVP alone and cells that received the combination of RGD4C/AAVP and LLnL. It is important to note that LLnL is considered to be a weak inhibitor of proteasomes compared to MG132. For example, transduction of human keratinocytes with a rAAV‐2 in the presence of LLnL did not show any significant difference in transgene expression (Braun‐Falco et al., 2005). Additionally, transduction of human primary endothelial cells with rAAV‐7 and rAAV‐8 was much more pronounced in the presence of MG132 than with LLnL (Denby et al., 2005). Finally, in our present study, the effective LLnL doses used to improve the efficacy of AAVP were dramatically higher than that of MG132.

Our results showing that MG132 increases tumor cell killing by RGD4C/AAVP were very interesting. We additionally showed that MG132 improves efficacy of targeted tumor transduction by RGD4C/AAVP *in vivo* after intravenous administration to tumor‐bearing mice. This is important because the intravenous route of administration is clinically utilized for both localized and metastatic disease. Moreover, proteasome inhibitors are being considered as anti‐cancer drugs in clinical trials for the treatment of cancer patients (Mujtaba and Dou, 2011; Park et al., 2008); thus, our data here together provide a foundation for using proteasome‐inhibiting drugs in combination with RGD4C/AAVP as a dual systemic antitumor therapy in cancer patients.

## 5 Conclusion

Our studies report a number of novel findings illustrating the proteasome as a significant barrier to targeted gene delivery by the RGD4C/AAVP in cancer cells *in vitro* as well as in tumors *in vivo*. Furthermore, we confirm that using RGD4C/AAVP in combination with a proteasome inhibitor is more efficient in killing cancer cells than either therapy alone. Proteasome‐inhibiting drugs should be considered for future clinical applications of targeted systemic gene therapy with AAVP in cancer patients.

## Conflict of interest

The authors declare that they have no conflict of interests to disclose.

## Supporting information



The following is/are the supplementary data related to this article:

Supplementary Figure 1 Effect of MG132 on RGD4C/AAVP‐mediated gene transfer in HEK293 cells. Cells were seeded in 48‐well plates and transduced with RGD4C/AAVP‐GFP or RGD4C/AAVP‐Luc and control non‐targeted vectors in the presence of 1 μM, 2.5 μM or 5 μM of MG132. After 4 h, an equal volume of complete medium was added to the cells and incubated at 37 °C overnight. Transgene expression was assessed at day 7 post transduction. A) Fluorescent micrographs showing GFP expression in HEK293 cells transduced with GFP expressing vectors. B) Luciferase assay in HEK293 cells transduced with Luc expressing vectors; the results represent the average relative luminescence units (RLU)/1 μg protein of triplicate wells. All experiments were repeated twice, and data were normalized to non‐targeted vector. Shown are data from a representative experiment.

Supplementary Figure 2 Representative pictures of U87 glioblastoma cells showing enhanced cytotoxic gene therapy by RGD4C/AAVP in combination with MG132. U87 glioblastoma cells were transduced with RGD4C/AAVP‐HSVtk or control non‐targeted vectors expressing the HSVtk gene in the presence or absence of MG132. Then cells were treated with GCV (20 μM) at day 3 post vector transduction, and renewed daily. Images were taken at 72 h post GCV treatment.

Supplementary Figure 3 Distribution of RGD4C/AAVP particles in the absence and presence of MG132. Immunofluorescence staining of RGD4C/AAVP followed by confocal microscopic analysis. M21 melanoma cells were incubated in vitro with targeted RGD4C/AAVP or control non‐targeted vectors for 4 h in serum‐free medium in the presence or absence of MG132, followed by growth in complete medium. After 24 h, internalized RGD4C/AAVP particles were detected using rabbit anti‐M13‐phage primary and goat anti‐rabbit AlexaFluor‐594 secondary (red) antibodies. Representative single optical sections are shown.

## Supplementary data 1

### 1.1

Supplementary data related to this article can be found at http://dx.doi.org/10.1016/j.molonc.2012.08.001.
