# Supplementary figures and images for: Proteasome inhibition in cancer is associated with enhanced tumor targeting by the adeno‐associated virus/phage
Source: Mol Oncol. 2012 Aug 21;7(1):55–66. doi: 10.1016/j.molonc.2012.08.001 (PMC3553581; doi:10.1016/j.molonc.2012.08.001)

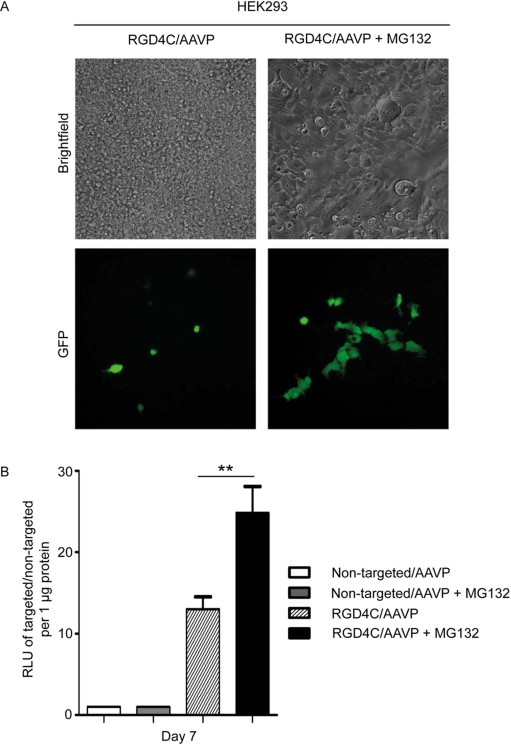

Supplement: Supplementary file 1 — Supplementary Figure 1 Effect of MG132 on RGD4C/AAVP‐mediated gene transfer in HEK293 cells. Cells were seeded in 48‐well plates and transduced with RGD4C/AAVP‐GFP or RGD4C/AAVP‐Luc and control non‐targeted vectors in the presence of 1 μM, 2.5 μM or 5 μM of MG132. After 4 h, an equal volume of complete medium was added to the cells and incubated at 37 °C overnight. Transgene expression was assessed at day 7 post transduction. A) Fluorescent micrographs showing GFP expression in HEK293 cells transduced with GFP expressing vectors. B) Luciferase assay in HEK293 cells transduced with Luc expressing vectors; the results represent the average relative luminescence units (RLU)/1 μg protein of triplicate wells. All experiments were repeated twice, and data were normalized to non‐targeted vector. Shown are data from a representative experiment. [file MOL2-7-055-s001.jpg]

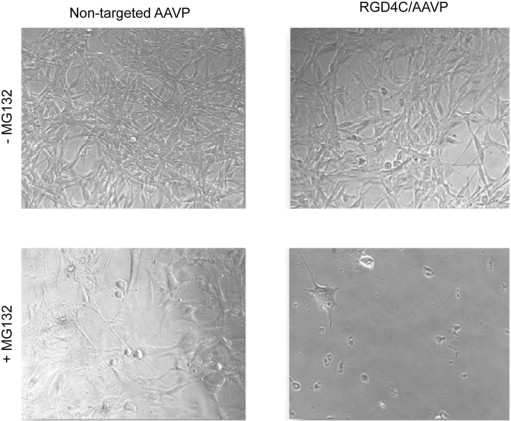

Supplement: Supplementary file 2 — Supplementary Figure 2 Representative pictures of U87 glioblastoma cells showing enhanced cytotoxic gene therapy by RGD4C/AAVP in combination with MG132. U87 glioblastoma cells were transduced with RGD4C/AAVP‐HSVtk or control non‐targeted vectors expressing the HSVtk gene in the presence or absence of MG132. Then cells were treated with GCV (20 μM) at day 3 post vector transduction, and renewed daily. Images were taken at 72 h post GCV treatment. [file MOL2-7-055-s002.jpg]

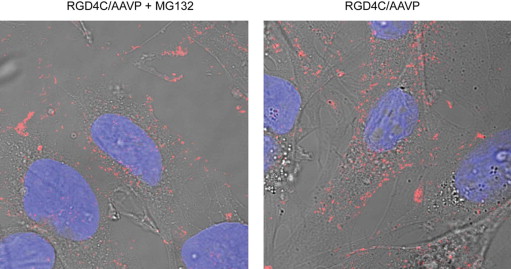

Supplement: Supplementary file 3 — Supplementary Figure 3 Distribution of RGD4C/AAVP particles in the absence and presence of MG132. Immunofluorescence staining of RGD4C/AAVP followed by confocal microscopic analysis. M21 melanoma cells were incubated in vitro with targeted RGD4C/AAVP or control non‐targeted vectors for 4 h in serum‐free medium in the presence or absence of MG132, followed by growth in complete medium. After 24 h, internalized RGD4C/AAVP particles were detected using rabbit anti‐M13‐phage primary and goat anti‐rabbit AlexaFluor‐594 secondary (red) antibodies. Representative single optical sections are shown. [file MOL2-7-055-s003.jpg]
